# A Comprehensive Study of the Potential Application of Flying Ethylene-Sensitive Sensors for Ripeness Detection in Apple Orchards

**DOI:** 10.3390/s19020372

**Published:** 2019-01-17

**Authors:** João Valente, Rodrigo Almeida, Lammert Kooistra

**Affiliations:** Laboratory of Geo-information Science and Remote Sensing, Wageningen University & Research, 6708 PB Wageningen, The Netherlands; rodrigo.almeida@wur.nl (R.A.); lammert.kooistra@wur.nl (L.K.)

**Keywords:** apple orchards, modeling and simulation, unmanned aerial vehicles, fruit ripeness, ethylene gas detection

## Abstract

The right moment to harvest apples in fruit orchards is still decided after persistent monitoring of the fruit orchards via local inspection and using manual instrumentation. However, this task is tedious, time consuming, and requires costly human effort because of the manual work that is necessary to sample large orchard parcels. The sensor miniaturization and the advances in gas detection technology have increased the usage of gas sensors and detectors in many industrial applications. This work explores the combination of small-sized sensors under Unmanned Aerial Vehicles (UAV) to understand its suitability for ethylene sensing in an apple orchard. To accomplish this goal, a simulated environment built from field data was used to understand the spatial distribution of ethylene when subject to the orchard environment and the wind of the UAV rotors. The simulation results indicate the main driving variables of the ethylene emission. Additionally, preliminary field tests are also reported. It was demonstrated that the minimum sensing wind speed cut-off is 2 ms^−1^ and that a small commercial UAV (like Phantom 3 Professional) can sense volatile ethylene at less than six meters from the ground with a detection probability of a maximum of 10%. This work is a step forward in the usage of aerial remote sensing technology to detect the optimal harvest time.

## 1. Introduction

Sustainable agriculture is a top priority for all the governments and nations worldwide. Our population is growing fast, and our resources are getting more scarce each day. By 2050, our population will reach nine billion, requiring crop production to double in order to meet food demands [1].

An efficient way to increase the upcoming demands is to avoid fruit spoiling during the harvesting. Immature fruits result in poor quality and are subject to mechanical damage, and overripe fruit results in a soft and flavorless quality, with a very short shelf-life. In general, if the harvesting is done too early or too late, physiological disorders in the fruits will be provoked with the consequence of a shorter shelf-life [2]. These issues becomes more relevant with international trade of fruit and vegetables is increasing, making the shelf-life become an important marketing tool [3]. Therefore, the Optimal Harvest Date (OHD) will dictate the resulting fruit yield.

The OHD is usually obtained from maturity indices that take into account fruit chemical composition, like total soluble solids or total acidity, fruit physical properties, like firmness or color, fruit physiological changes, like aroma and ethylene emission rate, and finally, chronological features, like the number of days after planting or blooming [4].

**Figure 1:**
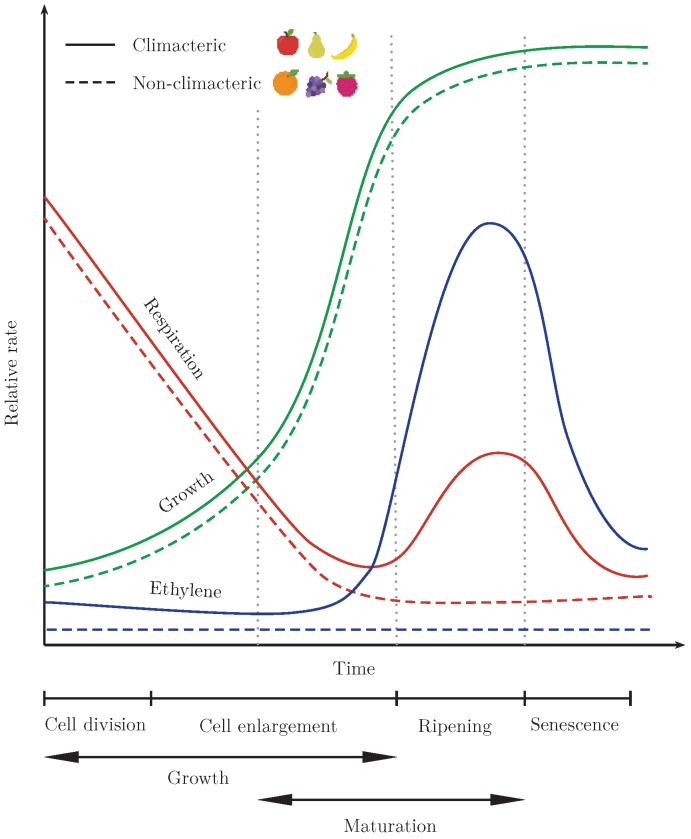
Relative rate of respiration, ethylene production, and growth in climacteric and non-climacteric fruits. Adapted from [5].

Fruits’ and vegetables’ lifespan can be broken down into three steps: maturation (i.e., increase in fruit size), ripening (i.e., increase in flavor), and senescence (i.e., tissue death) [3]. Fruits that ripen after harvesting are denoted as climacteric fruits [4]. For climacteric fruits, like apples, the optimal harvest date occurs when the pre-climacteric minimum happens, equivalent to the end of the maturation process or the beginning of the ripening process, as illustrated in Figure 1.

The fruits’ distinctive aromas are characterized by a wide variety of Volatile Organic Compounds (VOCs) that are released during their maturation process [6]. The VOCs can be detected using a single-gas sensor or an array of gas sensors (also known as an electronic nose) [7]. An important VOC that is associated with fruit ripening is ethylene [8].

Ethylene ($C_{2}H_{4}$) is a gaseous phytohormone that regulates several growth and development processes in plants. In climacteric fruits, ethylene production regulates processes like flesh softening, color changes, and aroma emissions during ripening [9]. Ethylene can be measured via gas chromatography techniques, electrochemical sensors, and optical sensors [10].

Most current destructive and non-destructive methods of assessing fruit maturity require the sampling of individual fruit in the field and in some cases a further assessment in the lab [8,11,12]. That process is both labor intensive, since it requires an operator to physically go to the field and sample fruits, and dependent on the individual fruits that are sampled. Using the electronic noses and gas measurements with the fruit in concentration chambers provides less noise and augments the ethylene signal substantially, but it requires time and manpower to harvest and analyze the fruit, and at the same time, it is a method that is highly reliable on the sampling scheme used for the fruit [13,14].

The increasing availability of UAVs is a potential solution to acquire remotely and quickly data on a plot of land without the manual labor that would be required traditionally. The land manager/owner does not have to survey the plot manually, but can deploy a UAV. There are several aerial remote sensing applications in agriculture that were successful reported as an important contribution and step forward in Precision Agriculture (PA) practices [15].

Using the combination of airborne and electronic nose technology to map ethylene concentration in the orchard might give important information regarding fruit maturity in a fast and more representative way, without the need for additional labor. To the author’s knowledge, no studies have been made so far regarding the potential limitations of this mapping application, but one could hypothesize that the sensitivity of the sensor and the atmospheric conditions during the measurements (i.e., wind speed and direction) are decisive.

Although plenty of research has been developed linking ethylene emission or VOC emission in apples to their maturity [8,16,17,18] and, in some literature, there are indications towards measuring ethylene in the field [11,19], to the authors’ knowledge, no work of this sort has been carried out. This work should, because of this, be considered as a first attempt at understanding the potential and the limitations of such measurements, creating with it a theoretical framework from which further work can be developed.

On the other hand, in air quality monitoring systems, some development has occurred considering mobile measurement platforms such as a UAV, especially when it comes to gas source localization and adaptive path planning for gas plume estimations [20,21]. Additionally, the optimal position of a gas sensor in the UAV has been studied using a simulation approach by [22]. Although some successful gas sensing experiments have been reported with the sensor pointing down [23,24], no literature was found regarding the challenges of measuring in an orchard environment, especially when it comes to the dispersion dynamics and its effect on the measurement process. Additionally, most of these works were performed using artificial gas sources that are easily modeled and do not take into account the complexities of a natural emission source such as apples.

The main goal of this work is to evaluate if ethylene produced by apple orchards can be sensed using an electrochemical sensor mounted on a UAV. The evaluation is made using a model-based approach to identify the most influential factors for detection, after which the model results are compared to measurements from a UAV-mounted electrochemical sensor flown over an experimental apple orchard.

## 2. Materials and Methods

### 2.1. Study Area

**Figure 2:**
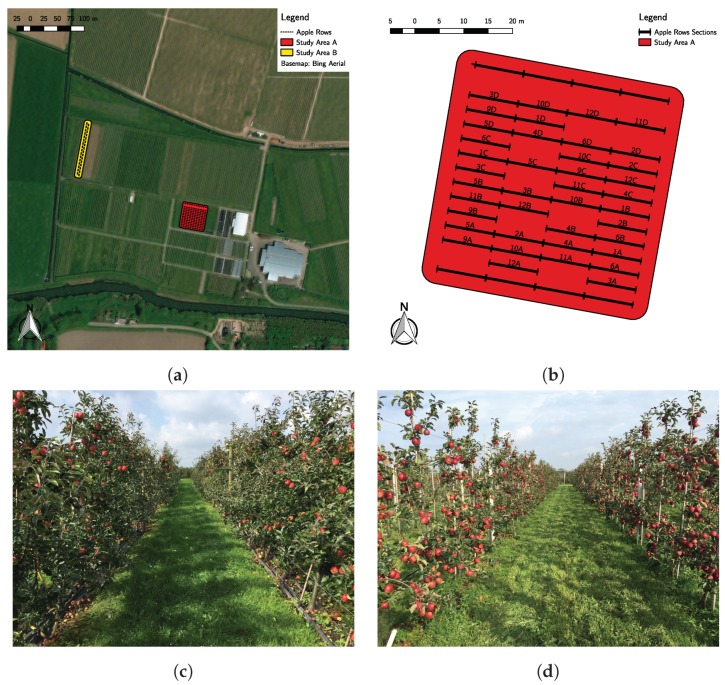
General and detailed map of the study area. (**a**) Map of the selected study areas in Randwijk (A and B). (**b**) Sections of apple lines used for fruit load assessment in Study Area A. Some lines show discontinuities since trees were removed in that section. (**c**) Junami and Golden Delicious cultivar. (**d**) Natyra cultivar.

The study area in which this research is based on is located in the Wageningen Plant Research for Flower bulbs, Nursery stock and Fruits in Randwijk, The Netherlands (see Figure 2a). A test plot of 0.17 ha of apple trees was selected (Study Area A). It had a length of 5 m in between tree rows and 1.1 m between trees in the row (5 × 1.1), which results in 14 lines of about 300 trees in the plot. Two apple (*Malus domestica*) cultivars are shown in Figure 2c: Junami and Golden Delicious (on the headers of each line for pollination purposes). Additionally, one other test plot was selected: Study Area B, which is a traditional apple orchard with 5 m between rows and 1 m between apple trees. The variety in this plot is Natyra. Only two lines were selected in Study Area B shown in Figure 2d.

### 2.2. Ethylene Flying Detector

The selected ethylene sensor was the Winsen ME4-C2H4, an electrochemical gas sensor, also referred to as a Taguchi gas sensor (TGS). According to the sensor specification sheet, it has a sensing range of 0–100 ppm of $C_{2}H_{4}$ and a response and recovery time of 100 s. Furthermore, the manufacturer indicates that the sensor has less than 10% of error.

**Figure 3:**
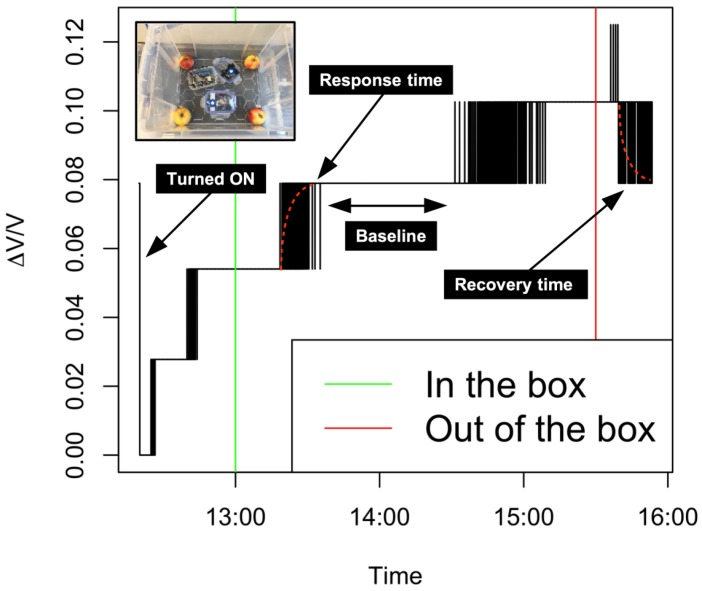
Tests conducted indoors in a sealed environment with an ethylene emission source (apples) that was placed in the box at the green line and removed at the red line.

In order to test both the ethylene sensor and the entire prototype, several preliminary experiments were conducted. With this, response times (amount of time it takes the sensor to detect the presence of ethylene) and recovery times (amount of time until the sensor signal returns to null after the ethylene source is removed) were tested. Figure 3 illustrates the results from the experiment in a controlled environment during four hours. The ethylene-sensitive sensor was placed inside a sealed plastic container of 40 cm × 50 cm × 40 cm with four *Junami* apples. After 3.5 h, the box was open, and after that, the sensor was placed outside the box.

**Figure 4:**
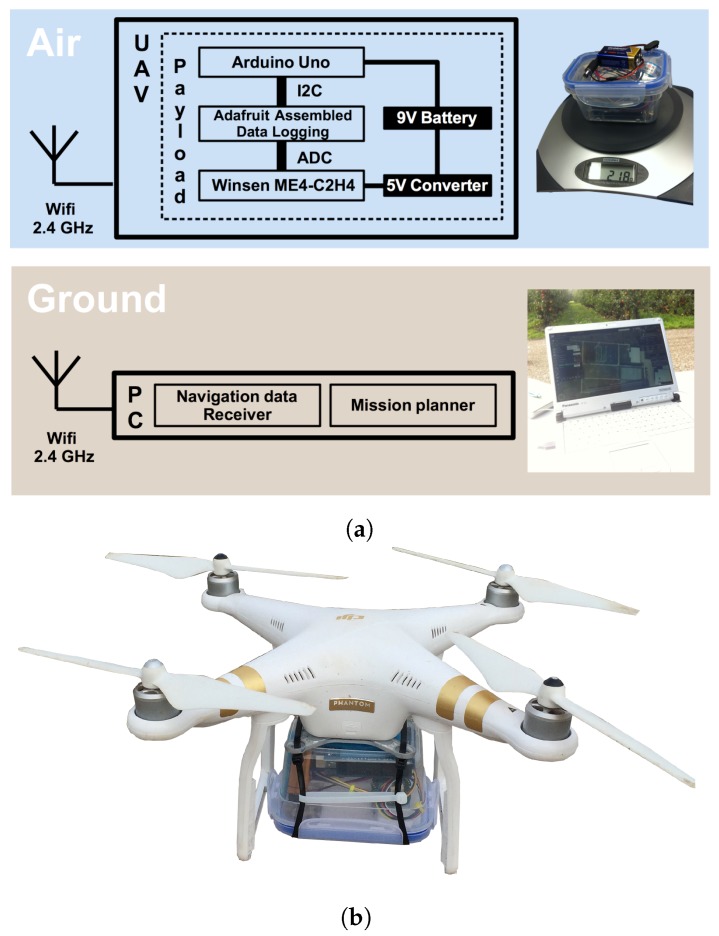
The ethylene flying-detector system: (**a**) air-ground system architecture and (**b**) Phantom 3 Professional (UAV) with the sensor prototype attached.

The UAV-based measurements were conducted with the Phantom 3 Professional. This is a quad-copter drone weighing 1280 g with approximately 23 min of maximum flight time designed primarily for photo and video capture applications. The default payload (an HD camera) was removed and replaced with the ethylene sensor, as illustrated in Figure 4. The maximum payload of the UAV is 300 g, and the total payload was 218 g (very similar to the default payload).

Additionally, the sensor prototype is equipped with a memory card when the device is on, with a configurable measurement frequency, were measurements are recorded with the respective time-stamp and output signal from the sensor. In these experiments, one measurement per second (1 Hz) was determined as the measurement frequency.

The complete remote sensing system design for detecting and measuring ethylene is illustrated in Figure 4, and it has three main components: electrochemical sensor, Arduino board, and battery. The system was composed of commercially-available materials and open source tools. Finally, it can be easily acquired with a cost of less than 1000 Euros.

## 3. Determining the UAV Hovering Height

Understanding how the ethylene emission distributes above the orchard canopy is very important in order to define a starting sampling strategy. Determining the height above the orchard canopy where ethylene presents a higher concentration is not a trivial task mainly because there are several biophysical parameters such as the wind speed, temperature, and humidity. In this study, the wind speed (environment) and wind flow (UAV rotors) effect on the ethylene distribution was observed, while the temperature and humidity were omitted.

In order to determine the ideal sensing position, a modeling and simulation framework was developed to decrease the system deployment and testing times. Moreover, it allows a more reliable data acquisition by restricting the aerial sampling to areas within the orchard where a minimum ethylene concentration is expected. The modeling and simulation framework used GADEN, a gas dispersion simulation framework developed by [25], which is compatible with the ROS (Robot Operating System) [26].

### 3.1. Environment Wind Speed Modeling

Several parameters had to be obtained from the orchard field manager and from the research center where the experimental field is located in order to build the simulation workspace in GADEN. The parameters taken into account to simulate the ethylene distribution within the orchard when subject to wind were:

1. Ethylene emission (μ Lh^−1^ kg^−1^). Each apple in the tree can in principle be at a different maturation stage and, therefore, have a different ethylene emission rate corresponding with three maturity stages.
2. Fruit position (height (m), direction (°)). Each apple in the tree can be at a different position in the canopy.
3. Fruit load (kg). Each apple tree can have a different amount of fruit.
4. Wind speed (ms^−1^). In any given moment, the local wind speed and direction might vary. For this initial evaluation, two wind speed directions were considered.

**Figure 5:**
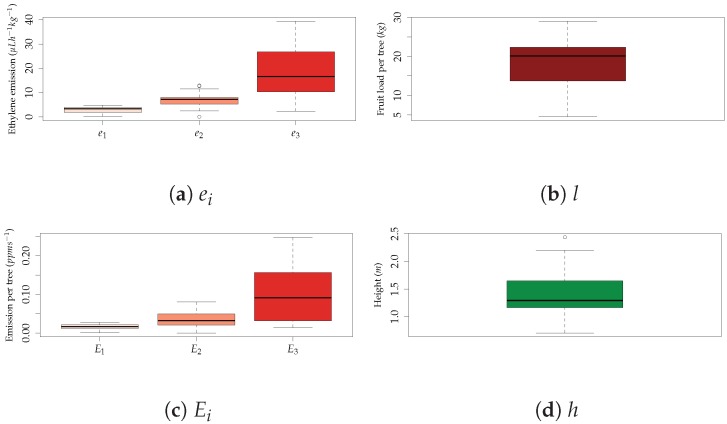
Distribution of the parameters used in the simulations: ${\left\{e,E\right\}}_{1}$, ${\left\{e,E\right\}}_{2}$, and ${\left\{e,E\right\}}_{3}$ stand for pre-climacteric, entering climacteric, and climacteric stages, respectively. Moreover, *l* stands for fruit load per tree and h for height.

These parameters were used to define an ethylene emission source for each tree represented in the simulation environment. Only one sample was taken per tree. Ideally, it would be possible to simulate each individual fruit on the tree canopy, but in this case, a simplification was performed using an artificial center for the total emissions of ethylene from a single tree. The distribution of the samples used in the simulations is illustrated in Figure 5.

This artificial center (P) can be described as the average position of the emission sources of the tree and is defined by a height (h) and direction in relation to the main stem (dir). This dir parameter in relation to the stem is defined in order to make the distribution of this parameter uniform, and therefore, the number of directions must be divisible by the amount of trees. In this case, six directions were defined, and each one was the sixth part of a circle, equivalent to 60 °.

**Figure 6:**
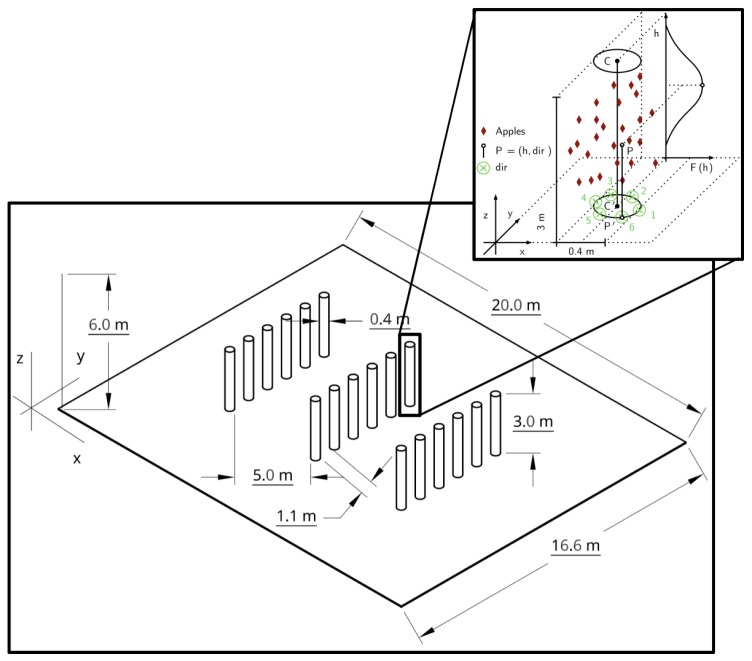
CAD model and respective parameters set in GADEN.

This simplification was applied mainly due to computational constraints. The simulator creates for each emission source a separate process, and for each process and time-step, the output is a simulation file of 90 MB. One can imagine that if each apple were simulated individually, the local memory of a standard computer would be very quickly surpassed. At the same time, according to our observations in the field, apples are usually clumped together in a branch, which means that the average distance between apples is in general small. Several branches can be further apart, but usually occupy one zone of the canopy. Figure 6 shows the orchard CAD model and the assumptions previously explained and used in the simulation process.

**Figure 7:**
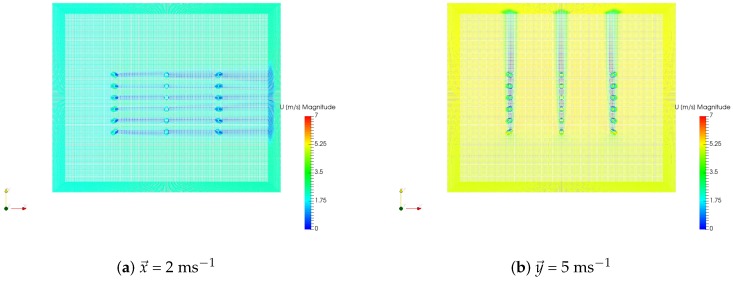
Wind flow simulations used as input for GADEN without considering the rotors’ airflow.

In the designed workspace, two environment inlets were set, the *x*-plane = 0 and the *y*-plane = 0. One of these inlets was chosen in order to simulate wind flow in a given direction: *x* for $\vec{x}$ and *y* for $\vec{y}$ (see Figure 7). The corresponding wind speed was assigned to this inlet, while the exact opposite plane (at the end of the environment) was set as a pressure outlet. All the other boundaries in the environment were set as walls with a slip setting. The computational fluid dynamics simulations were developed in SimScale, an online CFD software, with the recommended settings given in [25].

**Figure 8:**
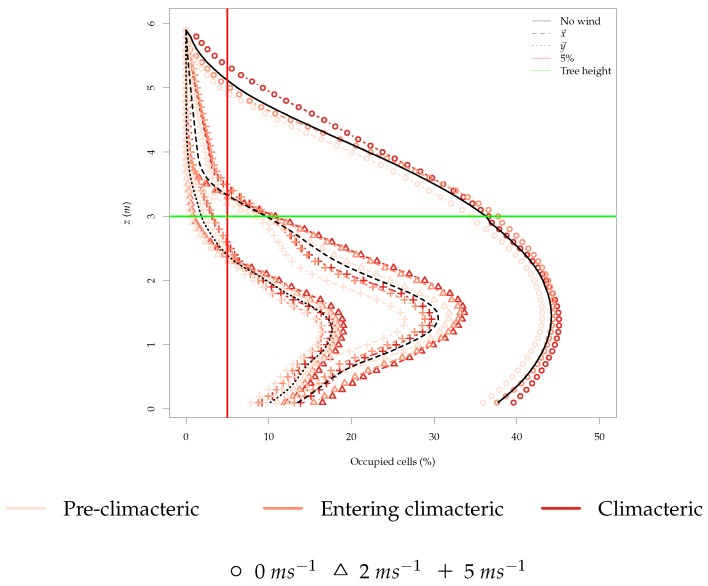
Percentage of occupied cells (cells with ethylene concentration higher than zero) in the environment across all time steps and simulations for the z-plane.

The number of ethylene-occupied cells in the environment is another important metric since it provides information on the probability of randomly finding an ethylene-filled cell. To get an understanding about which height is the most suitable to fly above the orchard, we must first look into the percentage of occupied cells with ethylene concentration above the canopy, as represented in Figure 8.

In almost all the simulations, less than 5% of the cells above the tree height were filled with ethylene. When wind speed was zero, there were more ethylene-filled cells above the tree height, but the majority of ethylene-filled cells can still be found under the tree height. It is also clear that the ethylene filled cells above the tree height had much lower ethylene concentration than the cells lower than the tree height. Therefore, the more likely place in the *z* axis to find ethylene-filled cells is between 1 and 2 m, where all simulations showed the biggest percentage of occupied cells.

**Figure 9:**
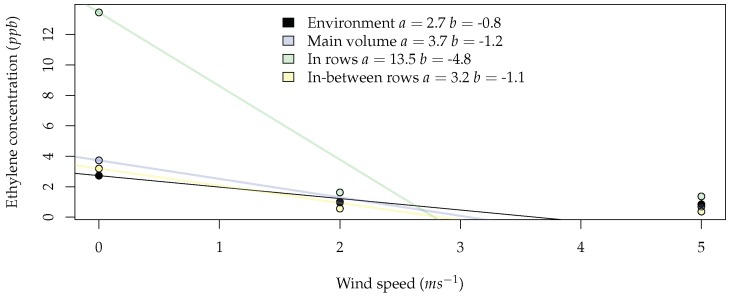
Relation between wind speed and average ethylene concentration in the four different zones. The colored lines represent the trend line for each zone, as given by the equation y=a+bx, where *b* is the decrease in average ethylene concentration (ppb) per additional unit of wind speed (ms^−1^).

To evaluate the impact of wind speed on the average ethylene concentration, Figure 9 was constructed. When looking at the environment, one can conclude that on average, a 1-ms^−1^ increase in wind speed results in a 30% decrease in average ethylene concentration. In the rows, the zone with higher average ethylene concentration, this decrease was 440%, while for in-between rows, this was only 110%. This difference is also accompanied by a very large difference in absolute ethylene concentration. This gives us an indication that choosing to sample in the rows might yield a higher concentration, but this measurement is very sensitive to the wind conditions.

From Figure 8 and Figure 9, it can be inferred that higher concentration levels of ethylene can be found below the trees and that the wind speed cut-off for the best practice is 2 ms^−1^. In the next section, the rotors’ airflow affect will be added to the environment to corroborate the results previously obtained omitting the rotors’ airflow.

### 3.2. Rotors’ Airflow Modeling

In order to simulate the effect of a UAV flying in the orchard, two different drone positions were considered: over the row (Position 1) and in between rows (Position 2). The drone over the row was positioned at 4 m, while the drone in between rows was positioned at 2 m. Only one wind scenario was considered for these simulations, $\vec{x}$ = 2 ms^−1^, in line with the results obtained in the previous section. This results in a total of six drone simulations, as exemplified in Table 1.

**Figure 10:**
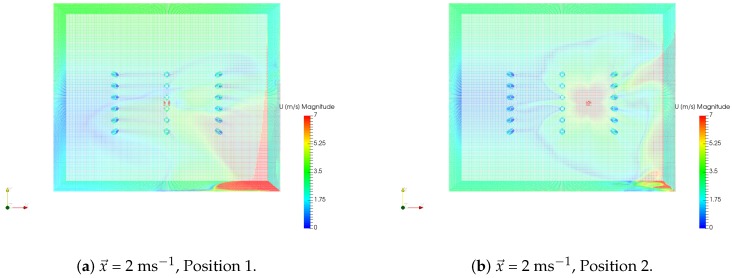
Drone wind flow simulations used as input for GADEN.

The wind flow caused by the rotors of the drone was modeled as four square air inlets with a given wind speed in the negative $\vec{z}$ direction. The squares had a width of 0.1 m, which is approximately the diameter of a single rotor in the Phantom 3 Professional. A relationship exists between the rotation speed of the propeller and the resulting wind speed generated, or thrust [27]. Taking the example of the Phantom 3 Professional in hovering flight in normal conditions, the rotors spin at around 8000 rpm [28], which results in an airflow of about 18 ms^−1^. This wind speed was assigned to the velocity inlets mentioned above. The resulting wind flow simulations used as input for the GADEN simulations are displayed in Figure 10.

**Figure 11:**
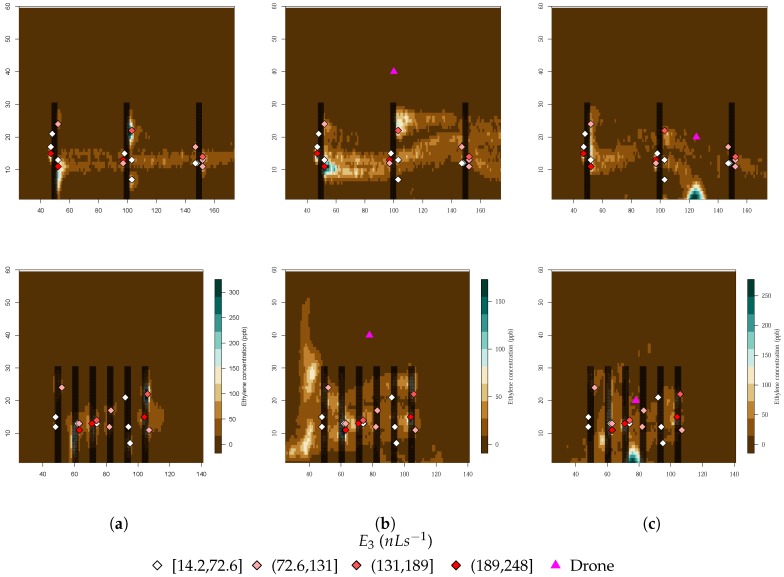
Maximum ethylene concentration in the xz-plane (top plots) and yz-plane (bottom plots) for the drone simulations in the climacteric stage: (**a**) omitting rotor wind flow; (**b**) Drone Position 1; and (**c**) Drone Position 2. The ethylene sources’ position and emission rate are also provided at the bottom.

The biggest difference between the ethylene concentration distribution with and without a drone appears to be the range of values that are present. When looking at the climacteric simulations with the drone in both positions, the maximum concentration was about 250 ppb, while without the drone, the same conditions yielded a maximum of 300 ppb. This range also decreased substantially with the height of the drone (Position 2 to 1), from 250 to 150 ppb, as Figure 11 clearly shows. There was also a gas concentration effect right under the drone position where it appeared that the wind displacement of the gas decreased.

**Figure 12:**
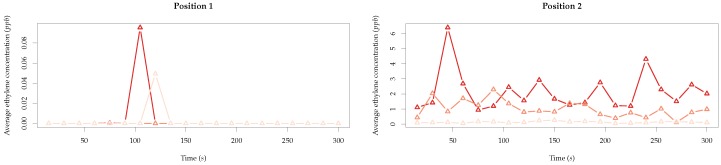
Average ethylene concentration across time in the vicinity of the drone position (±0.2 m in xyz) for Positions 1 and 2.

When looking at the immediate vicinity of the position of the drone, a clear difference was detected between Position 1 and 2, as Figure 12 illustrates. While no ethylene was detected around Position 1, at Position 2, in every simulated time step, ethylene was present. This is a consequence of the concentration effect mentioned above.

**Figure 13:**
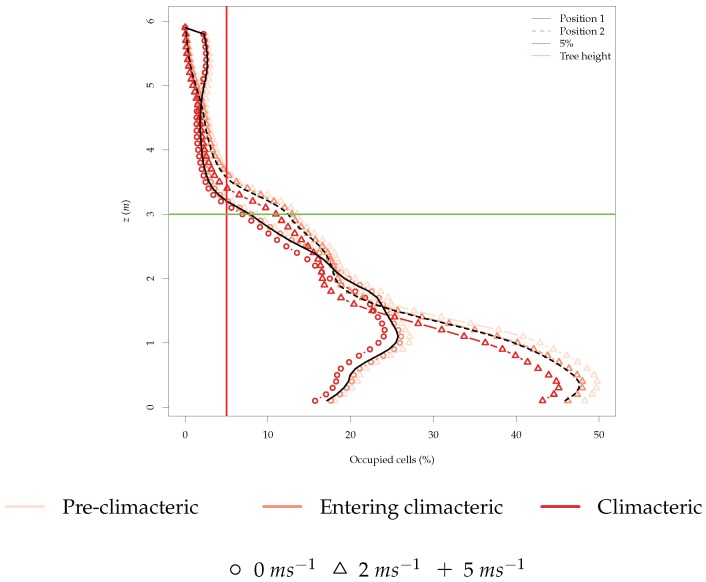
Percentage of occupied cells (cells with ethylene concentration higher than zero) in the environment across all time steps and simulations for the z-plane.

The distribution of the occupied cells in the environment was also very different, as Figure 13 illustrates. The percentage of occupied cells was in general lower due to the increase in average wind speed in the environment, and especially on the *z* axis, a compression of the occupied cells closer to *z* = 0 was visible, depending on the height of the drone, which further confirms the concentration of ethylene effect described previously. This compression results in a higher percentage of occupied cells closer to the ground.

In general, we can say that the drone flying overhead had two main effects: a decrease in average ethylene concentration in the orchard, directly correlated with the height of the drone (4 m caused more gas dispersion than 2 m) and a concentration of gas directly under the drone, close to the ground (an effect that was more discernible at a 2-m height). In general, the drone flying overhead at 4 m caused a decrease in average ethylene concentration of 95%, while at 2 m, a decrease in 90%.

## 4. Field Tests on the Orchard

The results obtained in Figure 3 provide evidence that both the wind speed and rotor wind flow played an important role in ethylene sensing. The behavior observed in the simulation is used now as the reference to define boundaries in the field tests, observe other measuring heights, and analyze the feasibility of this practice in the real orchard environment from an aerial mission perspective.

### 4.1. Sampling Scheme

**Figure 14:**
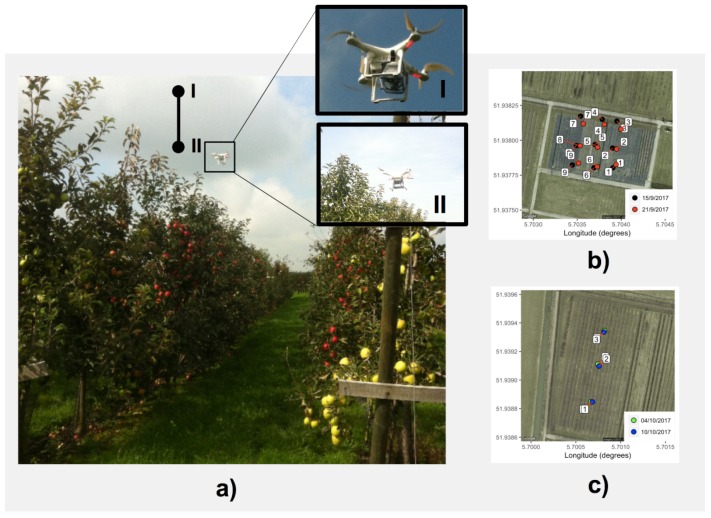
Hovering and sampling at two different position per experiment: (**a**) low and high heights within the orchard; (**b**) samples on Study Field A over the two days; and (**c**) samples on Study Field B over the two days.

In order to analyze the sensor functioning and detect ethylene in the selected plot on the ground and using a UAV, both a spatial and temporal sampling scheme was defined. The measurements with the UAV were conducted also per measurement point: at 6 m and 12 m during 120 s. This difference in sensing time has to do with practical constraints related to the battery life of the UAV. Please refer to Figure 14.

For Study Area A (Figure 2a,c), nine measurement points were selected for UAV-based measurements on two different days: 15 and 21 September 2017. These points were selected using a three by three grid in the plot and placing the point roughly in the center of each grid. Additionally, two UAV-based measurements were conducted also per measurement point: at 6 m and 12 m above the ground (3 m and 9 m above the canopy), with a sensing time of less than 3 min. This difference in sensing time has to do with practical constraints related to the battery life of the UAV employed. With a battery charge from the Phantom 3 Professional, Study Area A was sampled a maximum of nine times.

For Study Area B (Figure 2a,d), the same spatial sampling approach as Study Area A was used, but in this case, only two lines were taken into account. Three points were selected in the middle of these two apple lines, where measurements were taken as described before, with a different height in the UAV measurements: 4 and 6 m. These measurements were also performed on two different dates: 4 and 10 October 2017.

### 4.2. System Deployment and Sensitivity Tests

A weather station adjacent to the plot was used to provide real-time atmospheric measurements of wind speed and direction, air temperature, and air moisture during the sampling period on the different dates. The average wind speed on 15 September was about 3 ms^−1^, while on 21 September, it was about 3.2 ms^−1^. The average wind speed on 4 October was about 5 ms^−1^, while on 10 October, it was about 3.9 ms^−1^. Furthermore, the first harvesting day for Study Areas A and B was, respectively, 28 September and 10 October.

**Figure 15:**
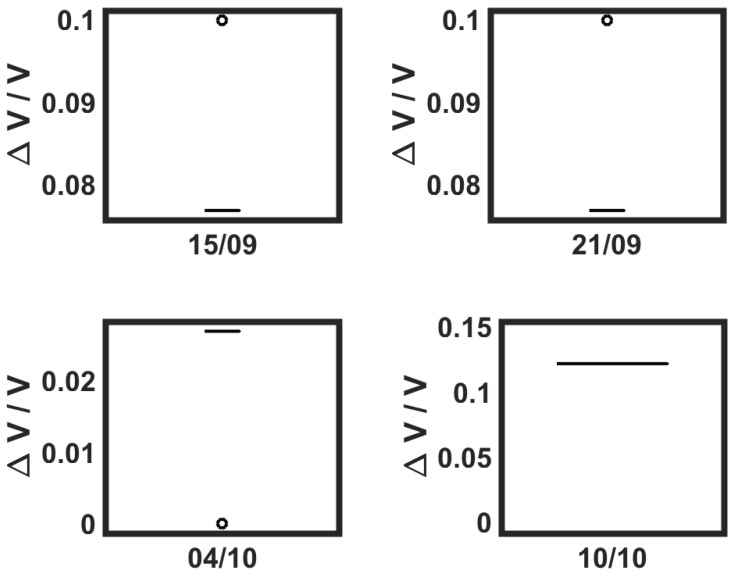
Sensor voltage output for aerial measurements on different days.

The average measurements obtained per day are shown in Figure 15. Looking at the UAV-based measurements, no output was measured above 10% of the reference signal, thus achieving a maximum of 0.5 ppm (500 ppb). We can notice that there was no variation in the first two days of measurements (Study Field A). Nevertheless, there was an increase from the 4–10 October, which in this case suggested that there was more ethylene concentration on the second date (which was expected). The decrease in wind speed might also explain some of this variation from one date to the other.

**Figure 16:**
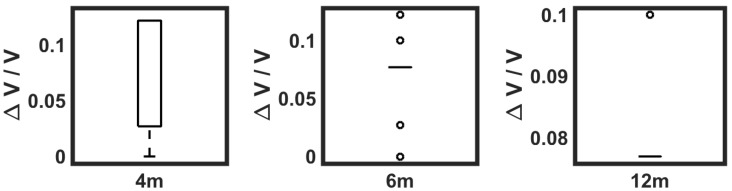
Sensor voltage output for aerial measurements at different heights.

When flying at different heights (see Figure 16), an interesting behavior was observed: the measurements performed at a higher altitude (12 m) showed no variation from the baseline; at 6 m, there were more outliers that indicated more detection peaks, and at only 4 m, some variation was actually detected.

## 5. Discussion

Although plenty of research has been developed linking ethylene emission or VOC emission in apples to their maturity [8,16,17,18] and in some literature there are indications towards measuring ethylene in the field [11,19], to the authors’ knowledge, no work of this sort has been carried out, and there is to date no investigation addressing flying ethylene-sensitive sensor systems.

The modeling part of this study has shown that several factors influence the ethylene emission from the apple trees: these will be discussed in more detail below. We included the main influencing factors in the model, but additional ones, like time of the day, might be important, as well. These should be evaluated in follow-up studies.

In this section, the lessons learned from this study and major outcomes will be summed up.

### 5.1. Wind Speed and Rotors’ Effect

The hypothesis that wind speed decreases the probability of ethylene detection was verified through simulation. Although, this supposition was expected, in the literature, there was not any discussion of the the maximum wind speed to ensure the minimum sensing. It was shown through simulation that the wind speed cut-off was 2 ms^−1^ (Figure 9).

Moreover, several authors discussed the rotor effect when using this sensor technology mounted in multi-rotor UAVs. It was stated that this is true, and there is a small margin left for detection that may be improved at a determinate hovering height (Figure 8).

### 5.2. Theoretical versus Practical Optimal Sampling Height

Simulation and practical results agreed that for wind speeds higher than 2 ms^−1^, there would be very few or almost no detections. The minimum wind speed recorded during the field campaign was 3 ms^−1^, and indeed, the sensor variation was very low.

The most important analytical outcome reinforced by the field tests was that flying lower would increase ethylene detection. Furthermore, flying close to or under the tree canopy gave a better result, although the margin for detection was limited, as shown in Figure 13. For the sake of the security and safety of the platform and the flying crew, the drone did not fly under the tree canopy, but it was stated that as the height decreased, more variation was observed (see Figure 16).

This study suggests that the UAV overflight should be performed at the lowest possible height to decrease the impact of the wind flow generated by the rotors on the ethylene distribution. However, further flying maneuvers should be explored when flying within or close to the orchard.

### 5.3. Discrete versus Continuous Sampling

It is important to note that the UAV measurements will only be considered during hovering flights over the determined measurement point. With that, the data acquired during the path of the UAV in the study area was not determinant, but it is an important point for further research considering moving and continuous measurements. However, the increased response time from the sensors (>90 s) should be taken into consideration in the sampling strategy to adopt.

### 5.4. Ethylene Detection over the Season and Inferring the OHD

The simulations showed that the range of ethylene concentration in an orchard was in the ppb range, and wind speed had a very big impact on this ethylene concentration. According to [29], the usual measuring range for VOCs starts at 100 ppb, and not very many sensors offer a sub-ppb range. If the state-of-the art of the technology does not provide a sensor with such characteristics, this might decrease the feasibility of this remote sensing strategy.

The observations when entering the climacteric stage were expected to be stronger than the ones observed in Figure 12. In order to determine the OHD, an ethylene increase during this stage was expected. Therefore, this strengths even more the idea that a sensor with more sensibility must be considered in future experiments.

### 5.5. Feasibility of Using Flying Ethylene-Sensitive Sensors

The challenges of measuring ethylene with a UAV in an orchard environment especially when it comes to the dispersion dynamics and its effect on the measurement process was not found in the literature.

On the other hand, in air quality monitoring systems, some development has happened considering mobile measurement platforms such as a UAV, especially when it comes to gas source localization and adaptive path planning for gas plume tracking [20,21]. The benefits of using the mobile platform for air quality monitoring and also for the purpose of this research are similar: they can offer high resolution sampling both at a spatial and temporal level at a low cost [23]. However, most of these works are performed using artificial gas sources that are easily modeled, and none takes into account the complexities of a natural emission source such as apples trees.

The optimal position of the gas sensor also has been discussed [22,23] and could have been a valuable reference in this study. However, in one work, the authors performed experiments indoors, inside a garage, and in the other work, the outcomes provided were very limited. Both authors suggested different sensors placements: pointing down separated from the main frame and on the top of the platform. In this study, the sensor was used pointing down because the frame of the UAV did not allow other configurations. Further, studies are needed to explore the position suggestion from the previous authors. Nevertheless, the simulations provided in this study reveal that higher concentrations values will be found mostly below the platform (see Figure 13).

### 5.6. UAV vs. UGV

The discussion of which mobile vehicle will perform better in a determinate agricultural management task is not new. In general, UAVs have more of a sensing role, like aerial surveying, where there is the need to increase spatial resolution; while Unmanned Ground Vehicles (UGV) have more of an actuation role, where there an action should be performed, such as mechanical weeding [30].

In this study, we were interested in a versatile platform that could carry different instrumentation and sample the orchard on different 2D and 3D positions. Moreover, while this could be achieved at different heights with the UAV, it would be limited to a static height with the UGV. Moreover, UAVs are considerable better than UGVs, as regards the price, maintenance, and portability. Summing up, they can offer high resolution sampling both at a spatial and temporal level at a low cost [23].

## 6. Conclusions

This is the first study to investigate the feasibility of using a flying ethylene-sensitive sensor systems in a fruit orchard only some days before being harvested. A simulated environment built from field data was used to understand the spatial distribution of ethylene within the apple orchard, to define the field sampling boundaries, and to evaluate how this influences the detection from a miniaturized sensor on a UAV. Finally, some preliminary tests in the orchard field were carry out to elucidate the sensor;s sensitivity and to contrast with the theoretical study.

The drone flight effect on the ethylene distribution was tested, and we concluded that flying at a higher altitude will cause more disturbance and lower the average ethylene concentration than flying lower. At the same time, at higher altitude, almost no ethylene is present in the vicinity of the drone. In general, the drone flying overhead at 4 m causes a decrease in average ethylene concentration of 95%, while at 2 m, a decrease of 90%. The detection margin is short and not sufficient to infer the fruit maturity, where increased variability over the season is expected. With these results, the issue of the measurement system sensitivity is further confirmed: a requirement for a sub-ppb ethylene sensor is clearly supported.

The use of a UAV to perform ethylene measurements in an uncontrolled environment such as an apple orchard still needs to be further explored, but it is suggested that future practices using this system are imminent with further research. The effect different UAV propeller spans on the intensity of dispersion of the gas and also detailed response models of different sensor models are, among others, pressing issues to be considered in the future.

References

## Figures and Tables

**Table 1:** Summary of drone simulator runs. The simulation number (#) will be used as a reference for naming each of these scenarios in the following sections.

#	Ethylene Emission	Wind Direction	Wind Speed (ms^−1^)	Drone Position
1.1	Pre-climacteric	$\vec{x}$	2	(10,7.8,4)
1.2	Entering climacteric			
1.3	Climacteric			
2.1	Pre-climacteric	$\vec{x}$	2	(12.5,7.8,2)
2.2	Entering climacteric			
2.3	Climacteric			
